# Supplementary material for: Notch2 controls non-autonomous Wnt-signalling in chronic lymphocytic leukaemia
Source: Nat Commun. 2018 Sep 21;9:3839. doi: 10.1038/s41467-018-06069-5 (PMC6155045; doi:10.1038/s41467-018-06069-5)
Supplement: Supplementary file 1 — Supplementary Figures and Tables [file 41467_2018_6069_MOESM1_ESM.pdf]

Supplementary Figures and Tables

Mangolini, Götte et al.

Notch2 controls non-autonomous Wnt-signalling in chronic lymphocytic leukaemia

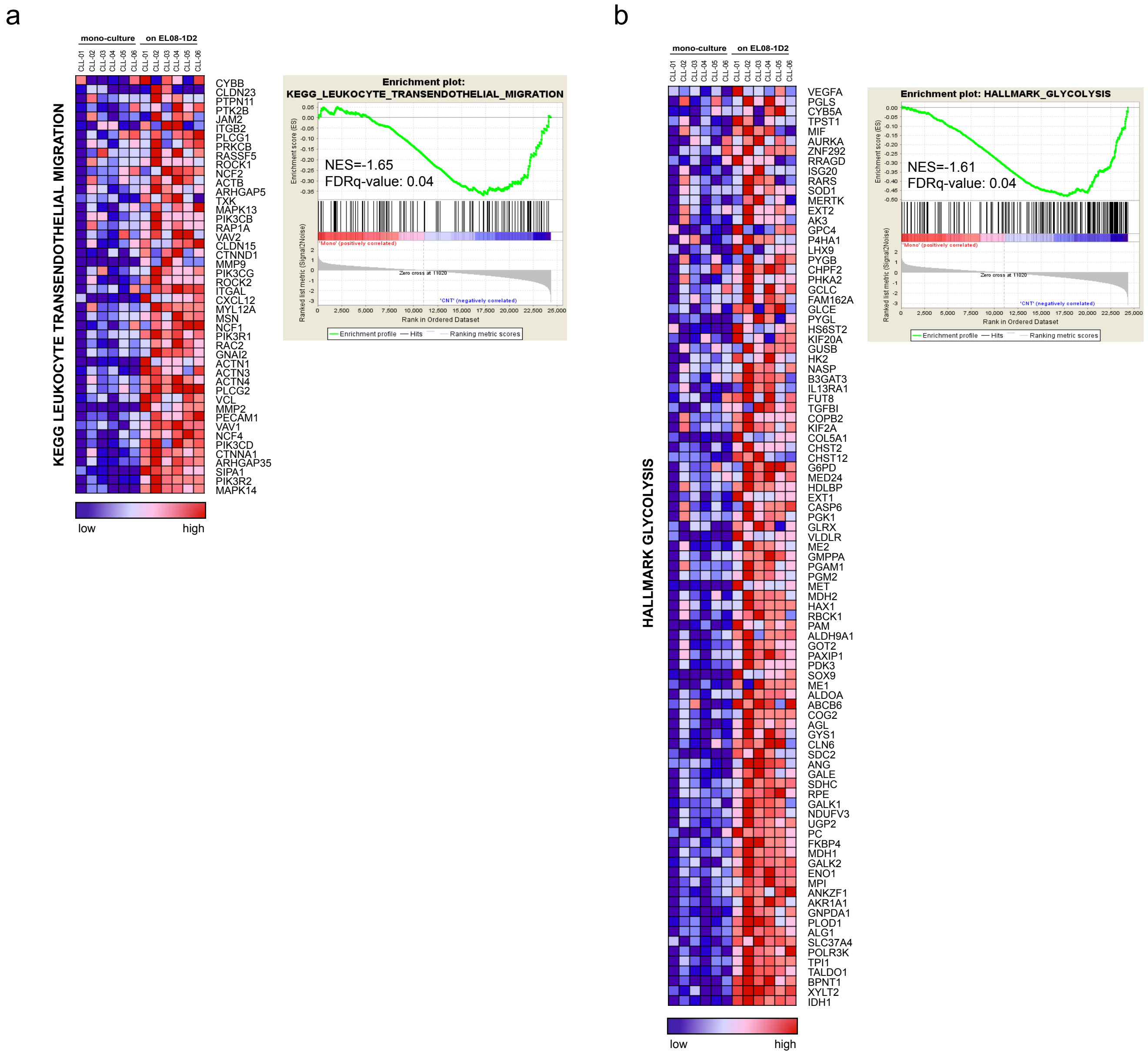

Supplementary Figure 1

a,b: The figure shows the enrichment plot and the heat-maps of gene sets relevant for several core pathways and altered following CLL-MSCs contact according to Gene set enrichment analysis (GSEA). All probe sets interrogating the respective target genes are given.

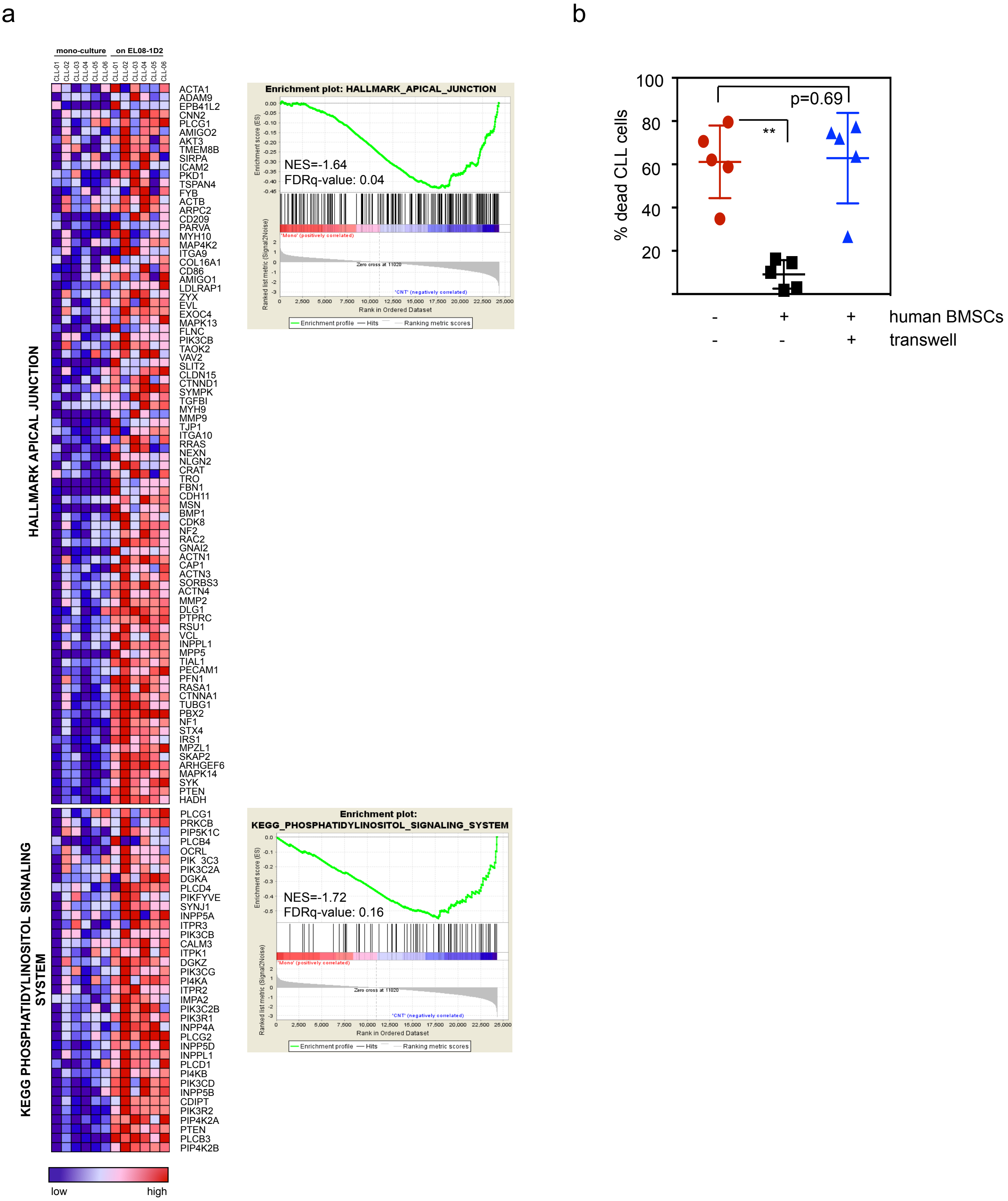

Supplementary Figure 2

a. The figure shows the enrichment plot and the heat-maps of gene sets relevant for several core pathways and altered following CLL-MSCs contact according to Gene set enrichment analysis (GSEA). All probe sets interrogating the respective target genes are given.

b. CLL cells were cultured in medium only (red circles) or on human BMSCs cells (black squares) for 5 days before analysing apoptotic cells by Annexin-V/DAPI staining. Transwells were used to disrupt direct cell-cell contacts (blue triangles). Error bars show mean ± SEM from 5 patients. **p < 0.01.

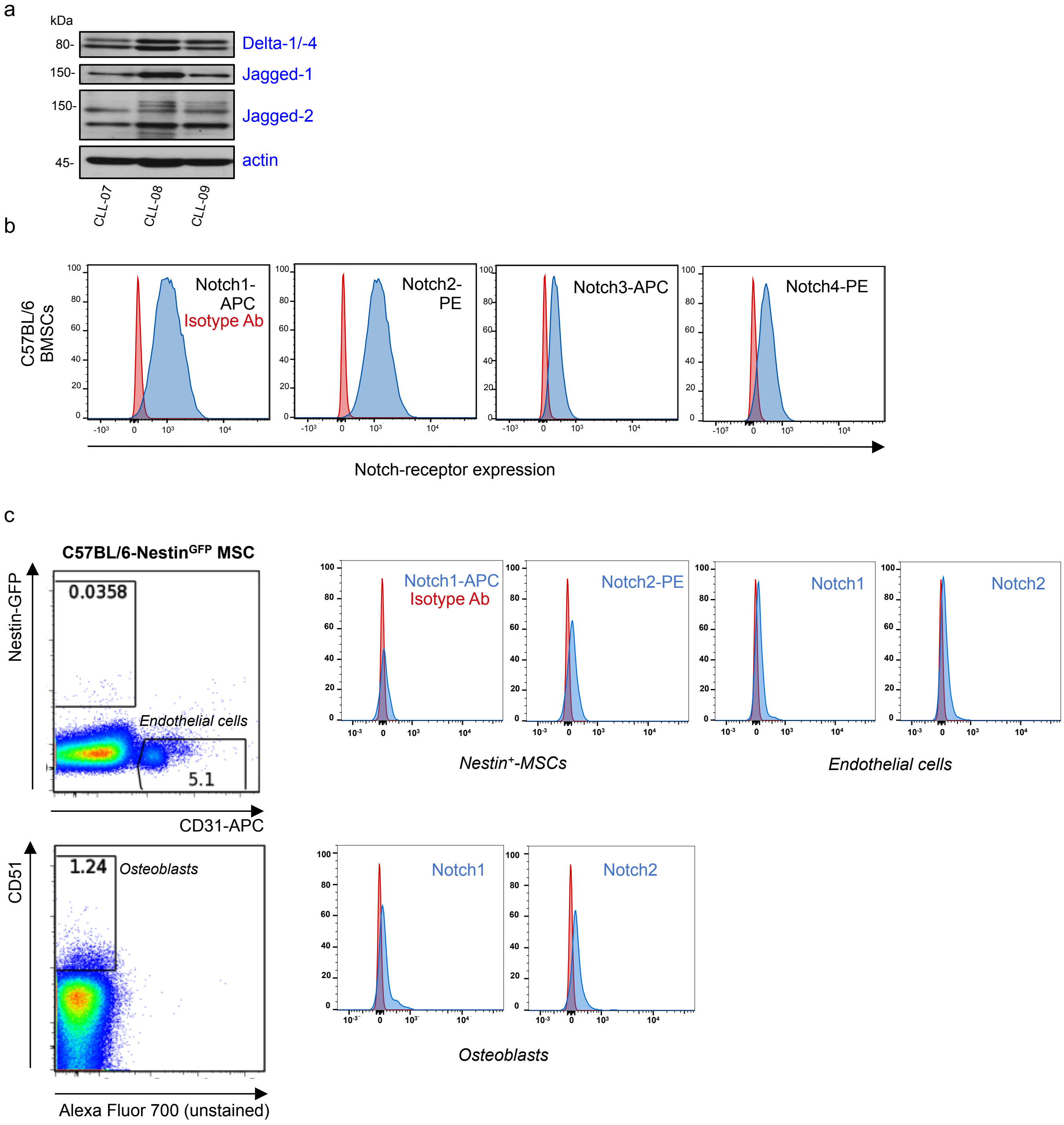

Supplementary Figure 3

a. Constitutive expression of the Notch-ligands Delta-1,-4, Jagged-1 and Jagged-2 were analysed in primary CLL cells obtained from 3 different patients by immunoblotting.

b. Expression of Notch1-4 in cultured primary MSCs derived from 8 weeks old C57BL/6 mice, assessed by flow cytometry.

c. Expression of Notch1 and Notch2 in primary MSCs derived from the bone marrow of 10-weeks-old C57BL/6 *Nestin*^{GFP} mice. Nestin-positive cells were identified by staining for GFP, endothelial cells by staining with CD31, osteoblasts were CD51 positive. Analyses of 3 additional mice revealed similar results. Isotype controls for Notch were used as negative control and performed on the bulk population.

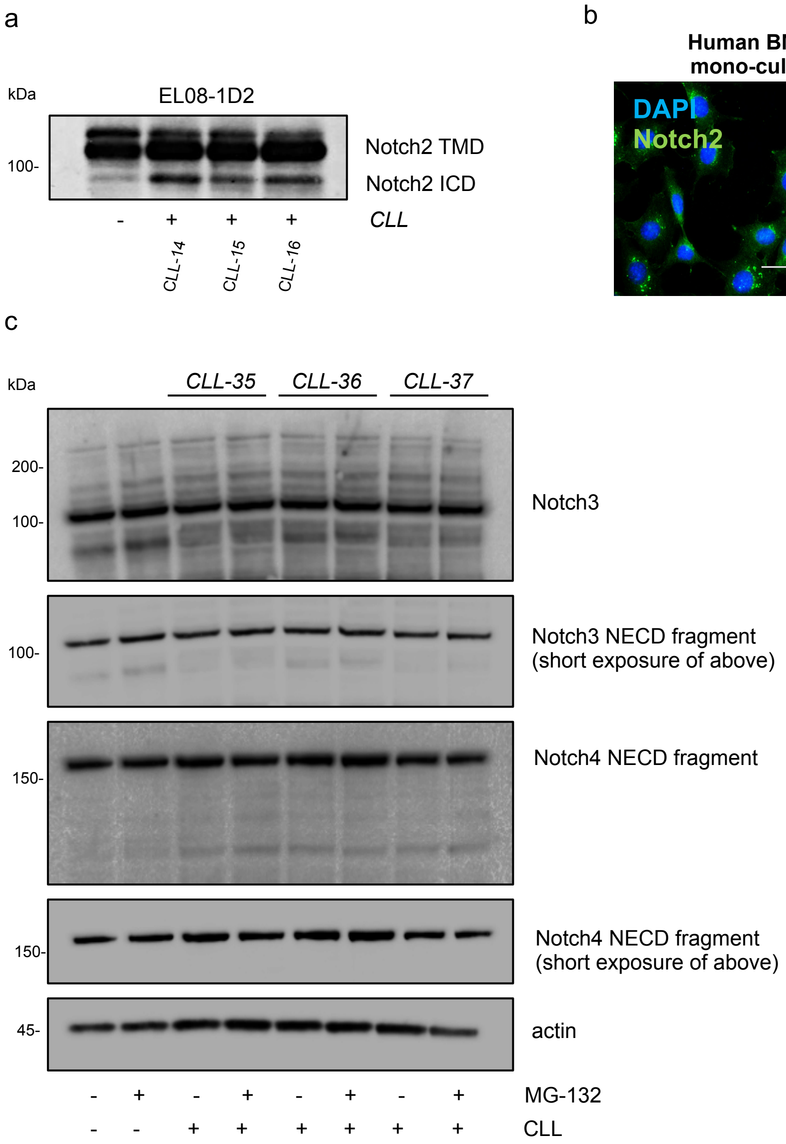

Supplementary Figure 4

a. Transmembrane-domain (TMD) and cleaved (ICD) Notch2 levels were analysed in EL08-1D2 cells and EL08-1D2 cells co-cultured with CLL primary cells.

b. Notch2 expression of human BMSC cells mono-cultured or co-cultured with primary CLL cells for 48 hours. One representative experiment out of three is shown. Scale bar = 100µm

c. Notch3-4 were analysed in EL08-1D2 cells and EL08-1D2 co-cultured for 48 hours with CLL primary cells obtained from 3 different patients.

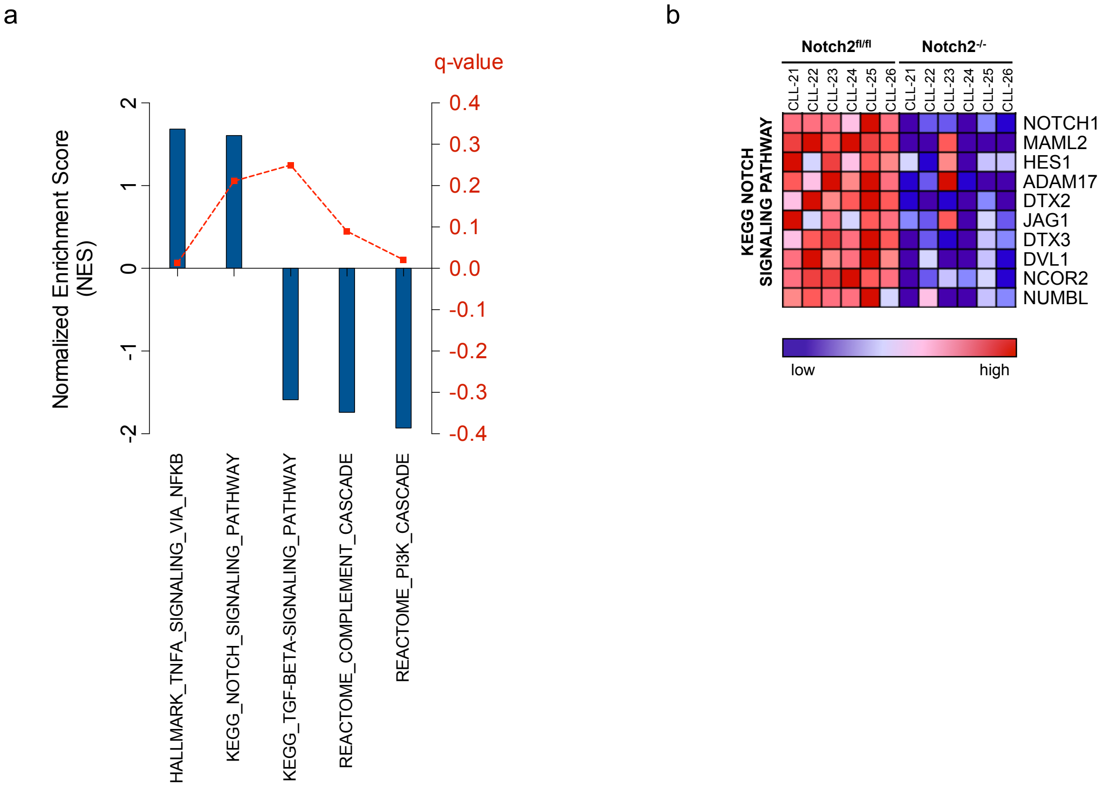

Supplementary Figure 5

a. GSEA comparing the expression of genes associated with the presence or absence of Notch2 in mono-cultured MSCs. Gene sets are listed in order of Normalised Enrichment Scores (left black Y-axis). FDR-q values for each gene set are indicated by the red-dotted line (right red Y-axis).

b. Heat map displaying the GSEA 'core' genes involved in Notch signalling following Notch2 ablation in MSCs co-cultured with primary CLL cells corresponding to the gene-set depicted in figure 3d.

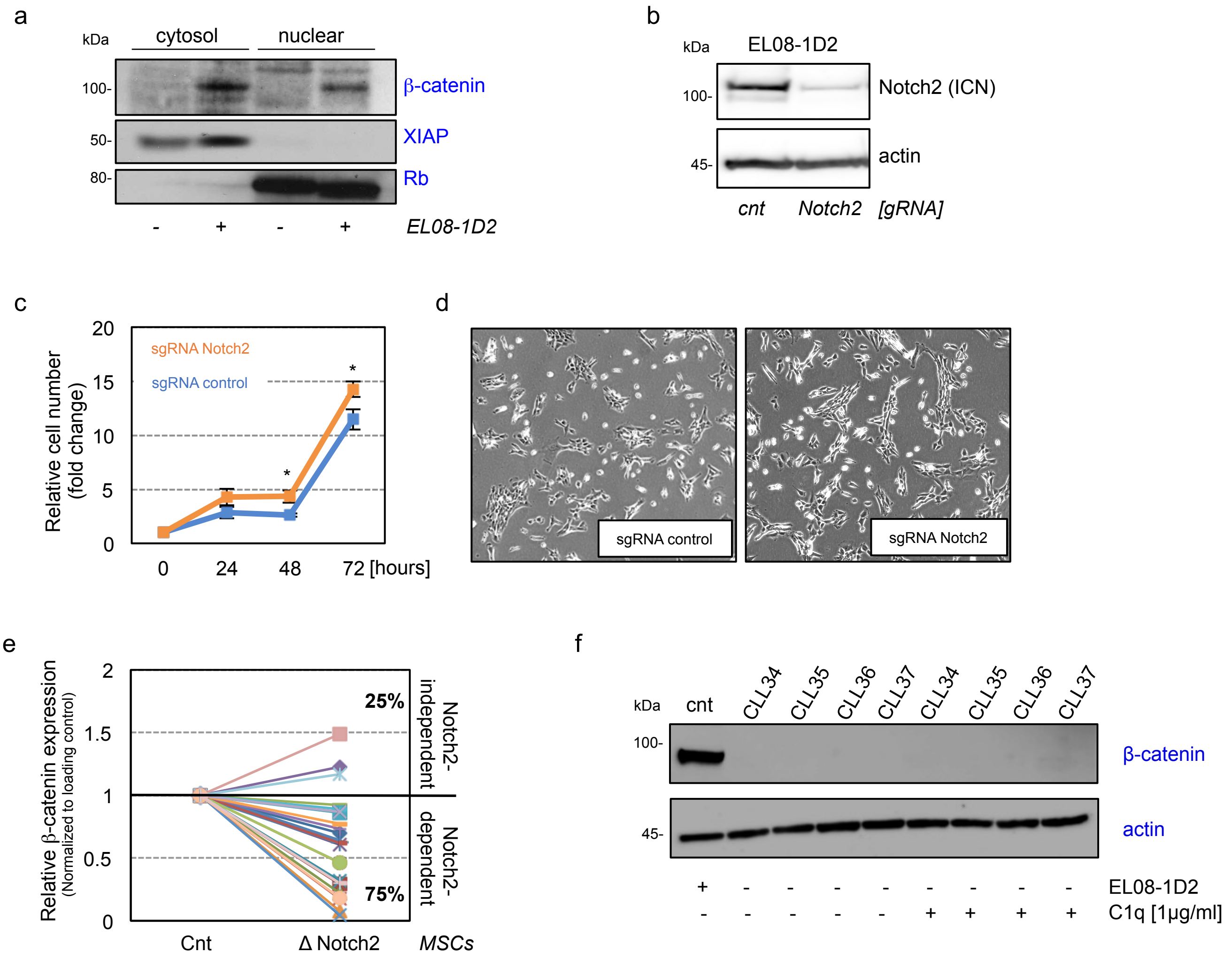

Supplementary Figure 6

a. Nuclear and cytoplasmic β-catenin levels were assessed in CLL cells derived from co-cultures with EL08-1D2 cells or from mono-cultures by cellular sub-fractioning and immunoblotting. Representative results from 3 different patients are shown. Nuclear expression was confirmed by co-expression of Rb.

b. Notch2 expression following CRISPR/Cas9 mediated ablation in EL08-1D2, assessed by immunoblotting.

c. EL08-1D2 cell proliferation was measured by cell count following CRISPR/Cas9 Notch2 deletion for 72h after cell plating. Results from 3 independent biological repeats are shown. Error bars depict SEM. $^*p < 0.05$.

d. Bright-field image of EL08-1D2 cells transduced with a sgRNA control or a Notch2 sgRNA 48h after plating.

e. Quantification of β-catenin levels in CLL cells, cultured on Notch2 proficient or deficient (using either Cre-mediated or Cas9-mediated deletion of Notch2) stromal cells. Immunoblots were quantified by using Image-J software. Expression was normalised to the expression of housekeeping proteins. 20 individual patients were analysed.

f. β-catenin expression in CLL mono-cultures after 24 hours in media supplemented with C1q (N=4).

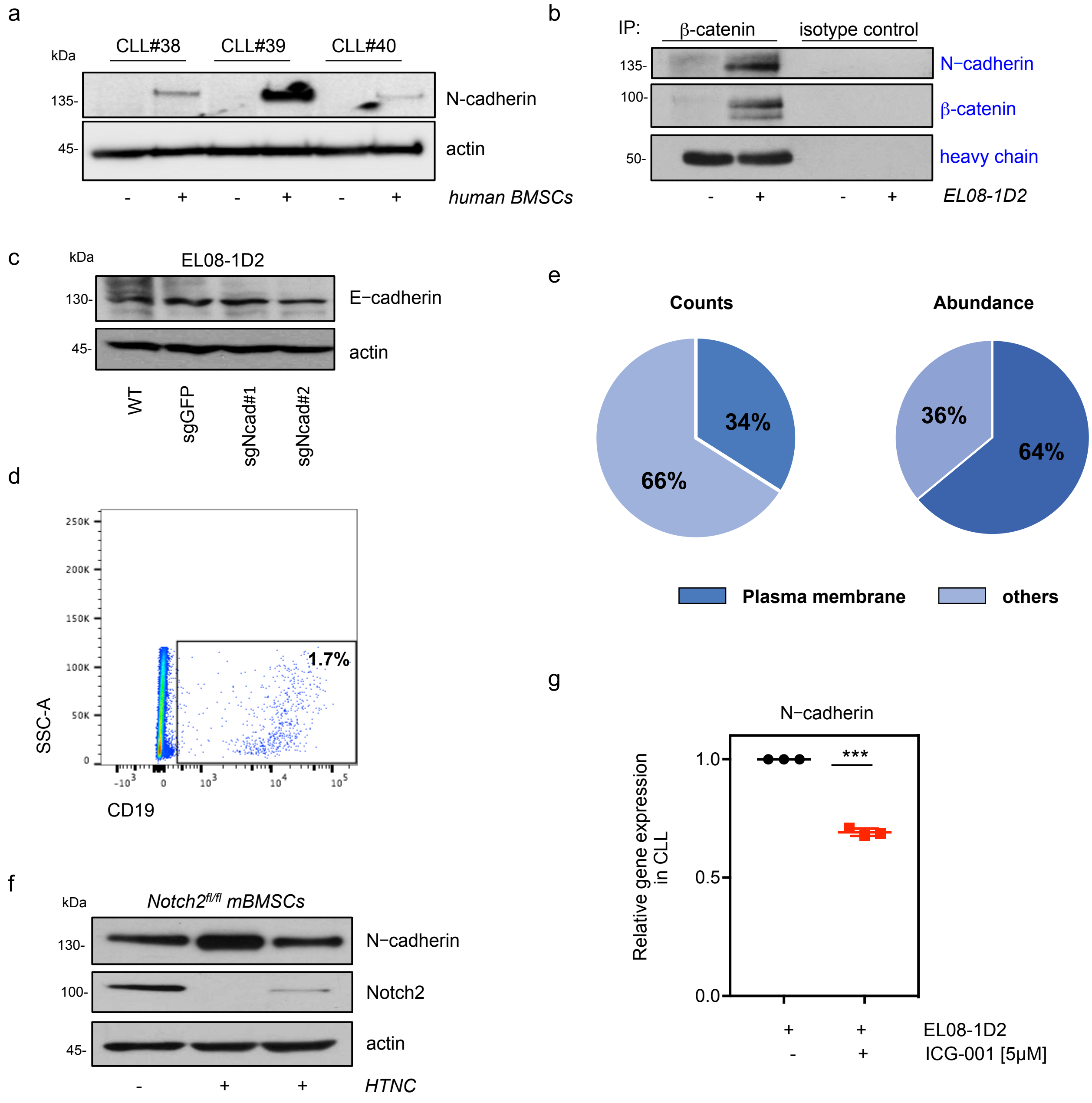

Supplementary Figure 7

a. N-cadherin expression in 3 primary CLL cells mono-cultured or co-cultured with human BMSCs for 24 hours.

b. β-catenin was immuno-precipitated from primary CLL lysates derived from mono-culture or from co-culture with EL08-1D2 cells and membranes probed with antibodies against N-cadherin. One representative experiment out of three is shown.

c. E-cadherin expression was evaluated by immunoblotting in EL08-1D2 cells in which N-cadherin expression was ablated using CRISPR/Cas9. Two different guide RNAs were used.

d. Flow cytometry analysis of $CD19^{+}$ cells contamination after aminooxy-biotin labelling of MSCs. The bulk of CLL cells were removed before labelling of MSCs.

e. Pie chart representing the total protein count and the total protein abundance of plasma membrane proteins following mass spectrometry analysis.

f. N-cadherin expression was evaluated by immunoblotting in $Notch2^{fl/fl}$ stromal cells following *in vitro* Cre-recombination. Two independent experiments are shown.

g. Quantitative reverse-transcription polymerase chain reaction analysis of N-cadherin mRNA expression in primary CLL cells 24 hours after co-culture on EL08-1D2 (black circles) and in presence of 5μM ICG-001 (red squares) normalized to expression in control cells (0μM ICG-001). Shown is the mean ± standard deviation of 3 independent experiments with individual primary CLL cells. ***$p < 0.001$.

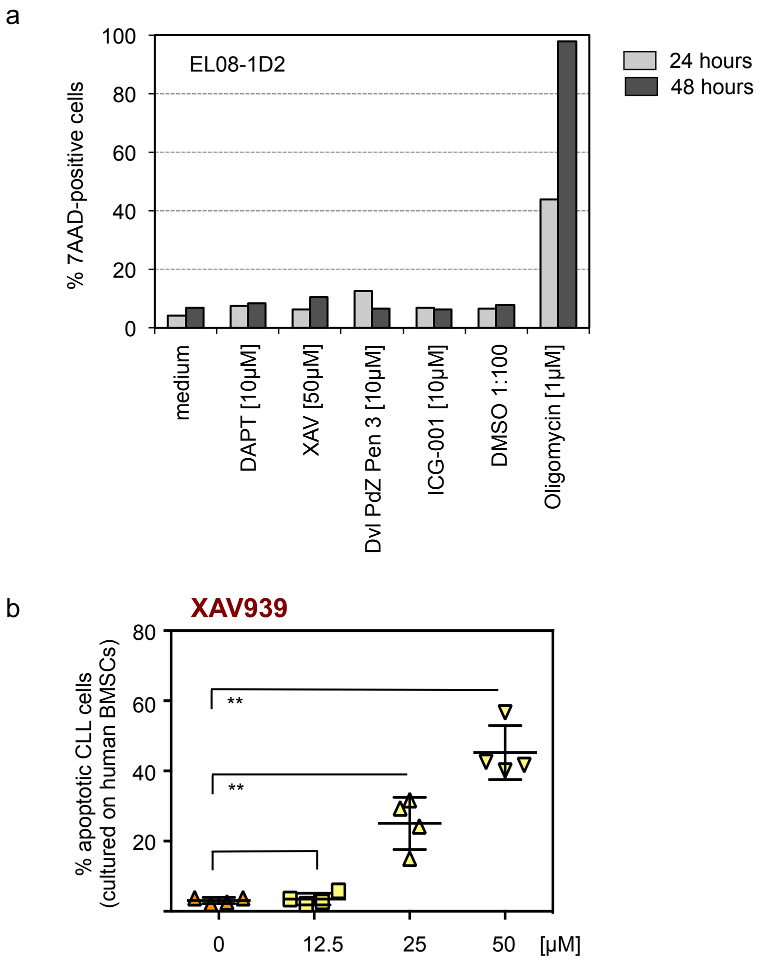

Supplementary Figure 8

a. Viability of EL08-1D2 cells, exposed for 48 hours to the inhibitors as indicated, was assessed by staining cells for 7AAD. Oligomycin treatment was used as positive control.

b. Percentage of apoptotic CLL cells co-cultured on human BMSCs and increasing doses of XAV939 were detected by flow cytometry and staining for Annexin-V/DAPI. Error bars show mean ± SEM from 4 patient samples. **p < 0.01.

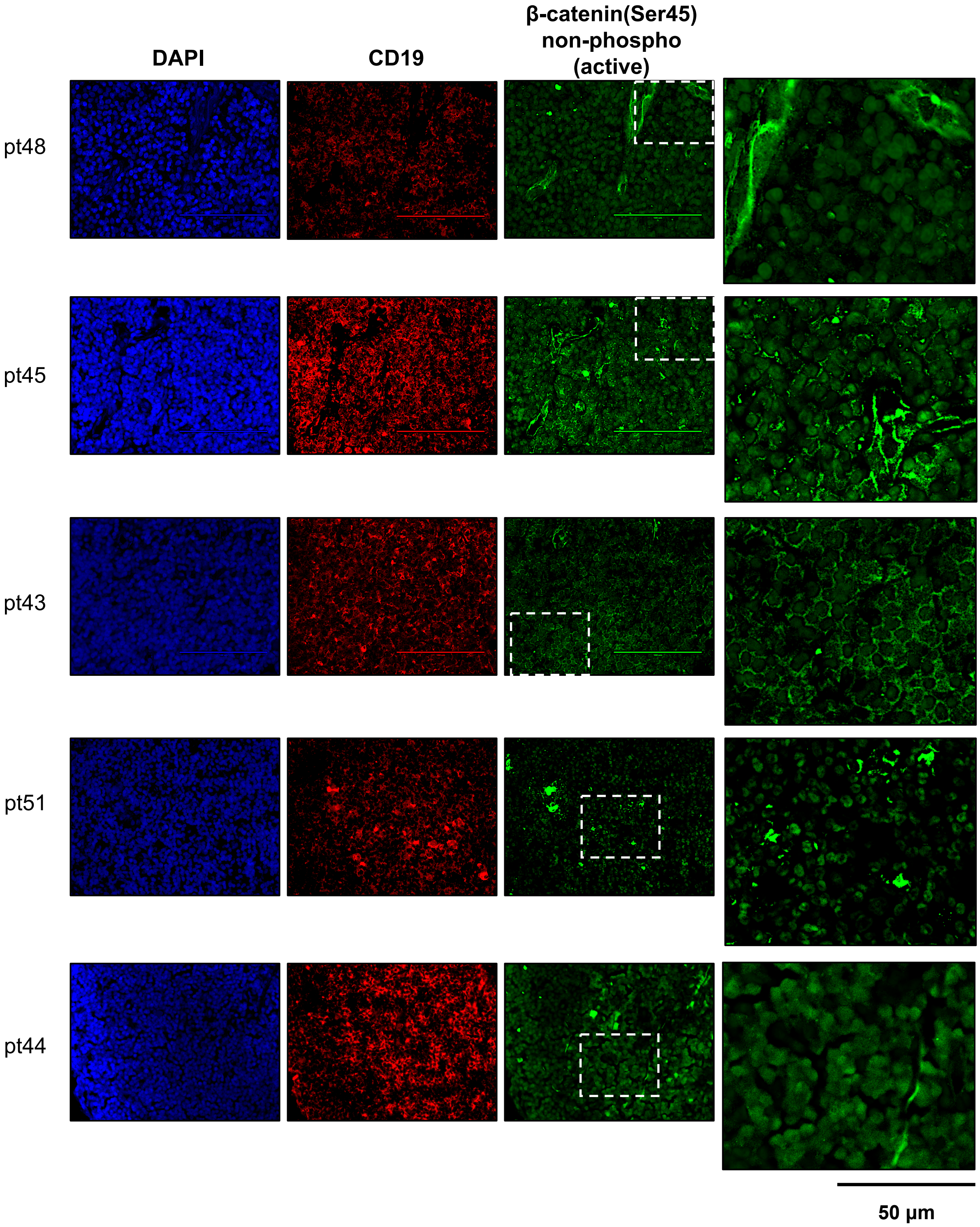

Supplementary Figure 9

β-catenin was assessed in lymph node sections of CLL patients by immunofluorescence microscopy. An antibody was used to specifically detect the active form of β-catenin. Scale bar=100µm. The white dotted box in the 3rd panel shows the tissue section enlarged and depicted on the right.

a

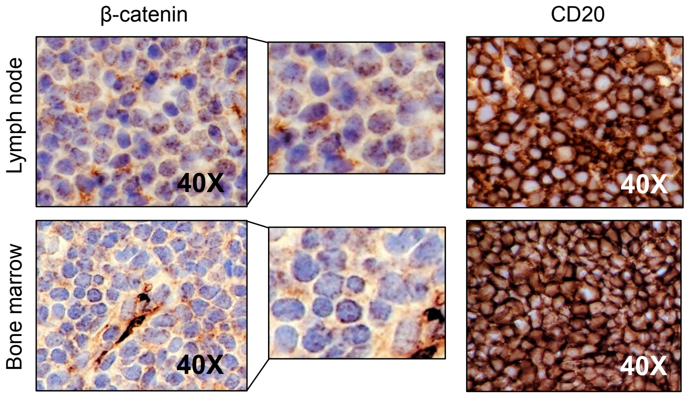

Supplementary Figure 10

a. Immunohistochemistry was performed on whole FFPE sections on both lymph node and bone marrow tissues from CLL patients. Six lymph nodes and 6 bone marrow trephine biopsies were examined with similar results

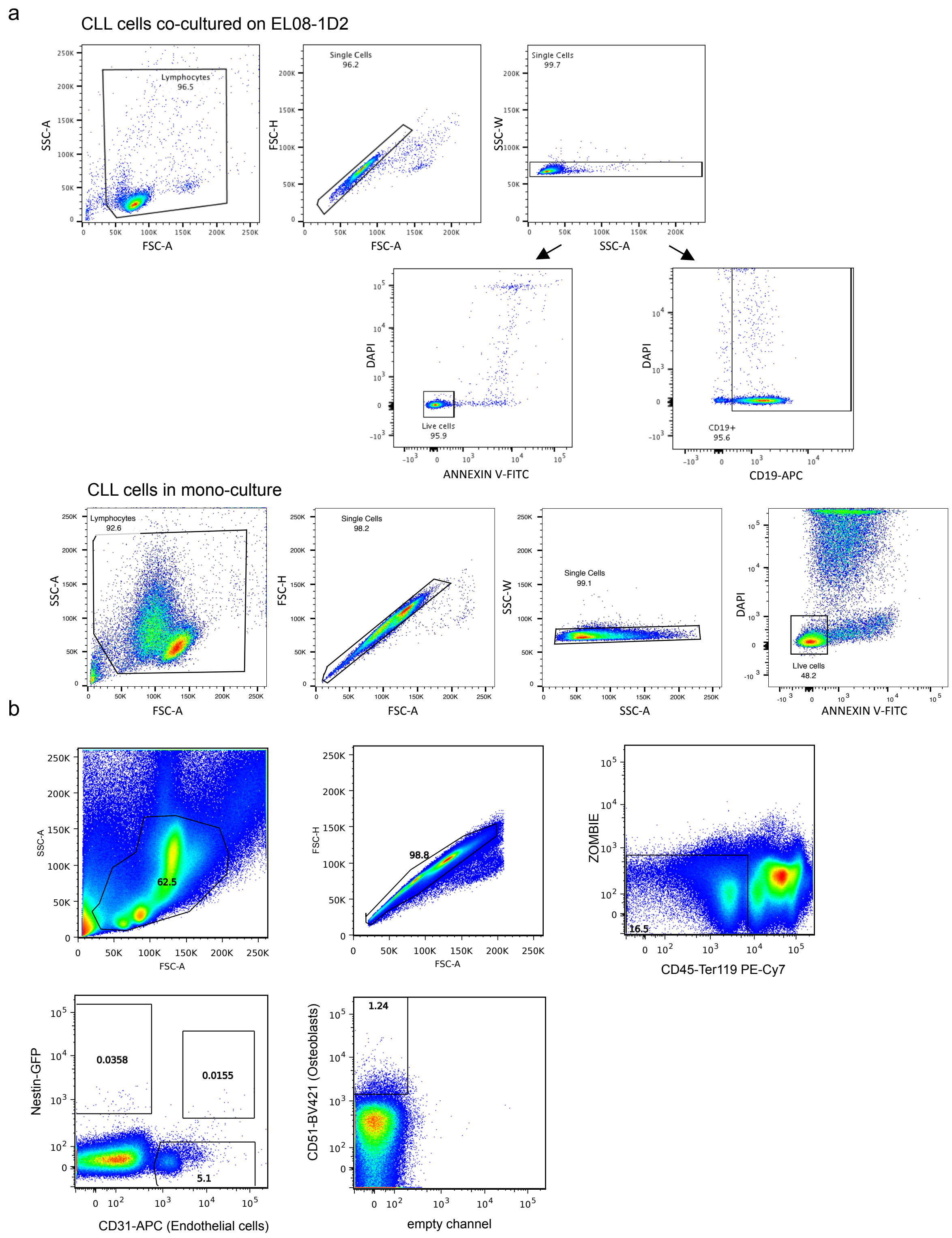

Supplementary Figure 11

a. Gating strategy for apoptotic CLL cells in mono-culture or in co-culture. SSC-A and FSC-A were used as the initial gate for cell debris and aggregates. In general, FSC-A/FSC-H followed by SSC-W/SSC-A was used to isolate singlets. DAPI was used to discriminate live from dead cells. Apoptotic cells were detected by staining for Annexin-V.

b. Gating strategy for bone marrow derived MSC subpopulations. SSC-A and FSC-A were used as initial gate for cell debris and aggregates. In general, FSC-A/FSC-H followed by SSC-W/SSC-A was used to isolate singlets. DAPI was used to discriminate live from dead cells. Nestin-positive cells were gated on CD45-negative cells.

a

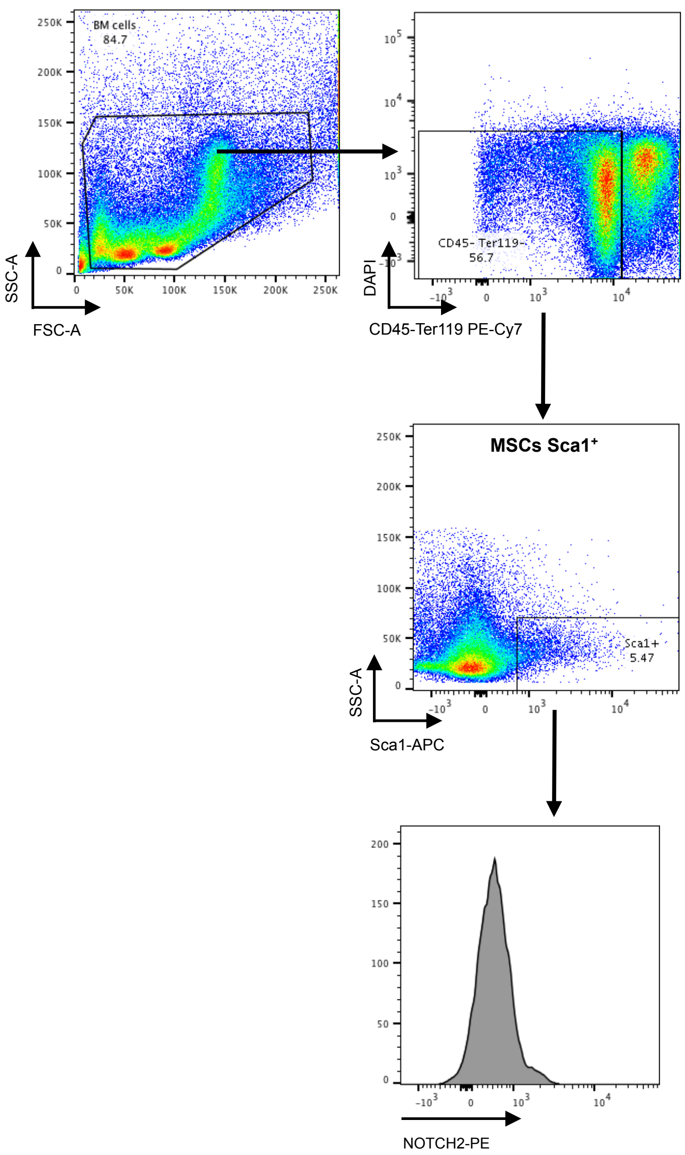

Supplementary Figure 12

a. Gating strategy used for the experiments depicted in figure 7b,c: A similar gating was applied as shown in supplement figure 11b with the exception that Sca-1 was used instead of Nestin/ CD31.

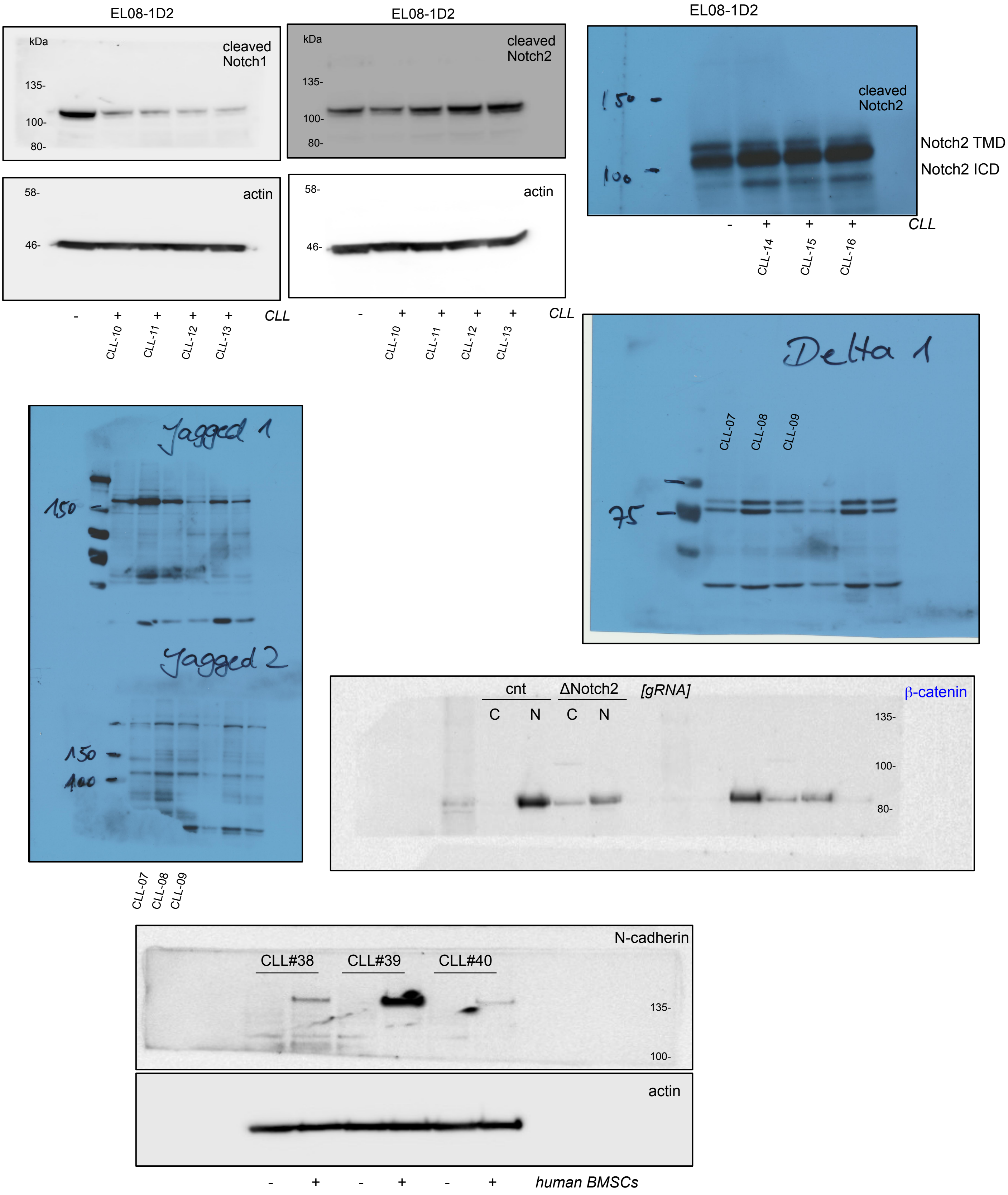

Supplementary Figure 13

Un-cropped western blots

Sex	Binet Stage	IGVH status/ FISH/ cytogenetics	ZAP70 expression	CD38 expression	Number of previous therapies	β-catenin dependency on stromal Notch2*
F	A	Not done	negative	negative	0	12%
F	A	Not done	negative	positive	0	10%
F	A	Not done	negative	positive	0	10%
F	A	Del 13q	Not available	negative	1	-88%
F	B	Not done	n.a.	n.a.	3	55%
M	A	Del 11q	n.a.	n.a.	3	0.2%
F	C	Del 13q	n.a.	negative	2	18.6%
F	B	Not done	n.a.	negative	0	-3.7%
F	C	Normal CLL FISH panel, p53 WT	n.a.	negative	1	46%
M	A	Not done	n.a.	negative	0	73%
F	C	Del 13q, Del 11q	n.a.	n.a.	1	88%
M	C	Del 13q	n.a.	negative	1	77%
F	A	Not done	n.a.	n.a.	0	-48%
F	A	Not done	n.a.	n.a.	0	-59%
M	A	Del 13q, Del 11q	n.a.	positive	1	-17%
M	C	Del 13q	n.a.	n.a.	1	30%
F	A	Not done	n.a.	n.a.	0	29%

ZAP70 and CD38 expression was determined by Flow Cytometry.

* β-catenin expression in CLL cells cultured on Notch2-deficient MSCs/ β-catenin expression in CLL cells cultured on Notch2-proficient MSCs expression was quantified by western blot and Image J software.

n.a.= data not available

Supplementary Table 1
Patient characteristics

Gene Title	Gene symbol	Fold change (log_2)	p-value
H-2 class II histocompatibility antigen, A-K alpha chain	H2-Aa	6.64	6.73113E-09
Histocompatibility 2, T region locus 24	H2-T24	5.25	3.177E-09
L-selectin	Sell	4.76	2.56346E-08
C-X-C chemokine receptor type 5	Cxcr5	4.74	2.95899E-08
H-2 class II histocompatibility antigen, E-D alpha chain	H2-Ea	4.37	2.09061E-08
H-2 class II histocompatibility antigen, A-F beta chain (Fragment)	H2-Ab1	4.35	1.40194E-08
C-X-C chemokine receptor type 4	Cxcr4	3.99	2.32547E-08
G-protein coupled receptor 183	Gpr183	3.93	3.32757E-07
Eukaryotic translation initiation factor 3 subunit A	Eif3a	3.85	4.32215E-08
H-2 class II histocompatibility antigen, I-A beta chain	H2-Eb1	3.57	3.62943E-08
Receptor-type tyrosine-protein phosphatase C	Ptprc	3.12	4.05805E-09
Neural cell adhesion molecule L1	L1cam	3.12	2.99476E-09
Ectonucleoside triphosphate diphosphohydrolase 1	Entpd1	2.92	5.74167E-09
Semaphorin-4D	Sema4d	2.64	2.54844E-07
Tyrosine-protein kinase Blk	Blk	2.59	7.69923E-08
Tetraspanin-33	Tspan33	2.37	8.23738E-06
Integrin beta-2	Itgb2	2.33	2.51935E-07
Ras-related C3 botulinum toxin substrate 2	Rac2	2.33	8.75252E-07
Proto-oncogene tyrosine-protein kinase LCK	Lck	2.14	4.82969E-06
H-2 class I histocompatibility antigen, K-D alpha chain		1.65	9.46389E-05
Integrin alpha-X	Itgax	1.63	0.000181204
H-2 class I histocompatibility antigen, K-K alpha chain	H2-K1	1.59	0.000144712
Semaphorin-4A	Sema4a	1.5	2.73726E-05
Cytochrome b-245 heavy chain	Cybb	1.4	4.85699E-05
Lysophosphatidic acid receptor 5	Lpar5	1.21	9.64464E-05

Supplementary Table 2
Plasma membrane proteins induced by CLL cells on EL08-1D2 cells

Gene Title	Gene symbol	Fold change (log_2)	p-value
Neurogenic locus notch homolog protein 2	Notch2	1.88	2.019E-06
Retrovirus-related Env polyprotein from Fv-4 locus	Fv4	0.95	0.000283718
Neuronal cell adhesion molecule	Nrcam	0.91	0.000937508
C3a anaphylatoxin chemotactic receptor	C3ar1	0.78	0.000421834
Uncharacterized protein FLJ45252 homolog		0.73	0.020251392
Complement decay-accelerating factor, GPI-anchored	CD55	0.71	0.001927835
C-C chemokine receptor type 1	Ccr1	0.66	0.005361613
Sodium-dependent lysophosphatidylcholine symporter 1	Mfsd2a	0.64	0.02517761
Clusterin	Clu	0.63	0.022191592
Traf2 and NCK-interacting protein kinase	Tnik	0.61	0.022085732
Glutamyl aminopeptidase	Enpep	0.6	0.002542121
Type-2 angiotensin II receptor	Agtr2	0.56	0.02609523
Hypermethylated in cancer 2 protein	Hic2	0.54	0.005111496
Killer cell lectin-like receptor 2	Klra2	0.53	0.034991894
Integrin beta-7	Itgb7	0.52	0.015223804
B2 bradykinin receptor	Bdkrb2	0.5	0.039249564
Inhibin beta A chain	Inhba	0.5	0.035432381
Calpain-6	Capn6	-0.41	0.036208618
Metalloprotease TIKI2	Trabd2b	-0.43	0.035024551
Collagen alpha-2(VI) chain	Col6a2	-0.44	0.043214805
Putative uncharacterized protein	2210010C04Rik	-0.45	0.04051605
Receptor-type tyrosine-protein phosphatase V	Ptprv	-0.49	0.028981233
Perilipin-4	Plin4	-0.5	0.027007517
Chondroitin sulfate proteoglycan 4	Cspg4	-0.52	0.004628809
Plexin domain-containing protein 1	Plxdc1	-0.53	0.013488852
Neurogenic locus notch homolog protein 1	Notch1	-0.54	0.039027808
Integrin alpha-11	Itga11	-0.57	0.010800793
PTB domain-containing engulfment adapter protein 1	Gulp1	-0.58	0.016305891
Leucine-rich repeat-containing protein 15	Lrrc15	-0.68	0.000826755

Supplementary Table 3
Plasma membrane proteins regulated by stromal Notch2 in CLL -EL08-1D2 co-cultures

Target	Clone<br>Cat. N.	manufacturer	Dilution	Figure
Delta	C-20<br>sc-377310	Santa Cruz Biotechnology	1:1000	S1a
Jagged-1	H-114<br>sc-8303	Santa Cruz Biotechnology	1:1000	S1a
Jagged-2	H-143<br>sc-5604	Santa Cruz Biotechnology	1:1000	S1a
N-Cadherin	H-63<br>sc-7939	Santa Cruz Biotechnology	1:1000	5a, 5b, 5d, 5i, 6c, S5a, S5b, S5f
HES-1	H-140<br>sc-25392	Santa Cruz Biotechnology	1:1000	6c
XIAP	2042	Cell Signaling Technology	1:5000	4f, S4a
β-Catenin	D10A8<br>#8480	Cell Signaling Technology	1:500	4a, 4b, 4c, 4d, 4e, 4f, 4g 4h, 4i, 4j, 5a, 5b, 5e, 6e, 8b, S4a, S4f, S5b
Non-phospho (Active) β-Catenin (Ser45)	D2U8Y<br>#19807	Cell Signaling Technology	1:1000 (WB)<br>1:100 (IF CLL)<br>1:50 (IF LN)	4d, 8a
Non-phospho (Active) β-Catenin (Ser33/37,Th41)	D13A1<br>#8814	Cell Signaling Technology	1:1000<br>1:100	4d
β-Actin	13E5<br>#4970	Cell Signaling Technology	1:10000	2,4,5,6,8,S2,S4,S5
NOTCH1	D1E11<br>#3608	Cell Signaling Technology	1:1000	2c
NOTCH2	D76A6<br>#5732	Cell Signaling Technology	1:1000<br>1:100	2d, 2e, 2f, 2g, 3b, 5d, S2e, S4b, S5f
GSK-3β	27C10<br>#9315	Cell Signaling Technology	1:2000	4g, 4h, 4j
p-GSK-3β Ser9	D85E12<br>#5558	Cell Signaling Technology	1:1000	4g, 4h, 4j
CD19	2E2B6B10<br>ab31947	Abcam	1:100	8a
NOTCH3	ab23426	Abcam	1:1000	S2f
NOTCH4	ab184742	Abcam	1:1000	S2f
Rb	G3-245<br>554136	BD Pharmigen	1:1000	S4a
NOTCH2 ICD	C651.6DbH N	Developmental studies Hybridoma Bank	1:1000	S2d
CAS9	7A9<br>MAC133	Merck Millipore	1:2000	5d

Supplementary Table 4
Antibodies used for immunoblotting

name	species	sequence 5`-3`
β-catenin forward	Homo sapiens	CCCACTGGCCTCTGATAAAGG
β-catenin reverse	Homo sapiens	ACGCAAAGGTGCATGATTTG
Gapdh forward	Homo sapiens	CCTGTTCGACAGTCAGCCG
Gapdh reverse	Homo sapiens	CGACCAAATCCGTTGACTCC
N-cadherin forward	Homo sapiens	AGGGTGGACGTCATTGTAGC
N-cadherin reverse	Homo sapiens	CTGTTGGGGTCTGTCAGGAT
C1qa forward	Mus musculus	CGGGTCTCAAAGGAGAGAGA
C1qa reverse	Mus musculus	TATTGCCTGGATTGCCTTTC
C1qb forward	Mus musculus	CAGGGATAAAGGGGGAGAAA
C1qb reverse	Mus musculus	TCTGTGTAGCCCCGTAGTCC
C1qc forward	Mus musculus	CTTCTGGCCCTACCACTCAG
C1qc reverse	Mus musculus	CTTCTGACCCTTGGGTCCTC

Supplementary Table 5
Primer sequence for qPCR analysis

Target	clone	Dilution	manufacturer	Figure
Notch1	HMN1-12	1:100	Biolegend	2b, S3b, S3c
Notch3	HMN3-133	1:100	Biolegend	2b, S3b
Notch4	HMN4-14	1:100	Biolegend	2b, S3b
Notch2	16F11	1:100	eBioscience	2b, 3b, 7b, S3b, S3c
CD45	30F11	1:200	eBioscience	7b, S3c
TER119		1:400	BD Biosciences	7b, S3c
CD31	MEC13.2	1:100	BD Biosciences	S3c
CD51	RMV-7	1:100	BD Biosciences	S3c
DLL1	MHD1-314	1:50	Biolegend	2a
Jagged-1	MHJ1-152	1:50	BD Biosciences	2a
Jagged-2	MHJ2-523	1:50	BD Biosciences	2a

Supplementary Table 6
Antibodies used for FACS analyses

name	species	sequence 5`-3`
SgRNA Notch2	Mus musculus	CGCCCGGTACTCACCGTGCG
SgRNA N-cadherin_1	Mus musculus	TGAAGCAAGGCCGCCAGAAG
SgRNA N-cadherin_2	Mus musculus	TTACAGCGCAGTCTTACCGA

Supplementary Table 7
sgRNA sequences
