# Supplementary material for: Notch2 controls non-autonomous Wnt-signalling in chronic lymphocytic leukaemia
Source: Nat Commun. 2018 Sep 21;9:3839. doi: 10.1038/s41467-018-06069-5 (PMC6155045; doi:10.1038/s41467-018-06069-5)
Supplement: Supplementary file 3 — Description of Additional Supplementary Files [file 41467_2018_6069_MOESM3_ESM.docx]

**Description of Additional Supplementary Files**

File Name: Supplementary Data 1

Description: Notch2-dependent, up-regulated genes in mBMSCs, activated by CLL cells

File Name: Supplementary Data 2

Description: Notch2-dependent, down-regulated genes in mBMSCs, activated by CLL cells
